# Septic shock in the era of precision medicine: rethinking fluids, vasopressors, and immunomodulation

**DOI:** 10.3389/fmed.2026.1909143

**Published:** 2026-09-23

**Authors:** Jialiang Du, Huizhen Feng, Shuangyong Dong, Chao Yang, Abdullah Al-Danakh, Siding Zhou

**Affiliations:** 1 Department of Critical Care Medicine, Aerospace Center Hospital, Beijing, China; 2 Department of Emergency, Hangzho Geriatric Hospital, Hangzhou, Zhejiang, China; 3 Department of Emergency, Hangzhou First People’s Hospital, Hangzhou, Zhejiang, China; 4 Amran Medical College, Amran University, Amran, Yemen

**Keywords:** septic shock, precision medicine, fluid responsiveness, vasopressors, immunomodulation, endothelial dysfunction, immune phenotyping, microcirculation

## Abstract

Septic shock remains a high-mortality syndrome despite advances in early recognition, antimicrobial therapy, source control, and protocolized resuscitation. A major limitation of current practice is that septic shock is often managed as a single clinical entity, whereas patients meeting the same diagnostic criteria may differ substantially in hemodynamic phenotype, endothelial injury, microcirculatory function, immune state, coagulation activation, fluid tolerance, and vasopressor responsiveness. These differences help explain why biologically plausible therapies frequently fail when tested in unselected populations. This narrative review synthesizes evidence supporting a precision-oriented approach to septic shock across three therapeutic domains—fluid therapy, vasopressor support, and immunomodulation—supported by two cross-cutting precision layers: biological phenotyping and bedside precision tools. We distinguish bedside clinical phenotypes from biological endotypes and explain how this framework can refine resuscitation decisions. Fluid therapy is reframed as a titrated intervention requiring assessment of both fluid responsiveness and fluid tolerance. Vasopressor therapy is discussed beyond a uniform mean arterial pressure target, with emphasis on timing, agent selection, tissue perfusion, and individualized weaning. Immune, endothelial, and coagulation phenotyping are presented as biological bridges between bedside physiology and targeted therapy, while immunomodulation is considered a promising but still investigational frontier requiring patient stratification. Finally, we propose a pragmatic precision-resuscitation framework that integrates lactate kinetics, capillary refill time, point-of-care ultrasound, congestion assessment, and immune/endothelial biomarkers where available. Precision care should not delay time-critical sepsis interventions; rather, it should mature protocolized management into a dynamic process of early treatment, repeated reassessment, and phenotype-directed adjustment.

## 1 Introduction

Septic shock remains one of the most challenging syndromes in critical care. Contemporary definitions conceptualize septic shock as a subset of sepsis characterized by circulatory, cellular, and metabolic abnormalities associated with a greater risk of death than sepsis alone (1, 2). Mortality remains high across healthcare systems, with substantial variation related to illness severity, infection source, comorbidity burden, access to intensive care, and timeliness of source control (3, 4). According to the Third International Consensus Definitions for Sepsis and Septic Shock, sepsis is defined as life-threatening organ dysfunction caused by a dysregulated host response to infection. Clinically, organ dysfunction is operationalized as an acute increase of at least 2 points in the Sequential Organ Failure Assessment score consequent to infection. Septic shock represents a more severe subset of sepsis and is clinically identified by a requirement for vasopressor therapy to maintain a mean arterial pressure of at least 65 mmHg together with a serum lactate concentration greater than 2 mmol/L despite adequate volume resuscitation. These operational criteria facilitate rapid recognition but do not capture the substantial hemodynamic, endothelial, metabolic, and immune heterogeneity that exists among patients meeting the same diagnostic definition (1, 2, 5). Current international guidelines standardize time-critical management through antimicrobial therapy, source-control planning, fluid resuscitation, vasopressor support, serial perfusion assessment, and repeated reassessment (6).

Despite these gains, a central problem persists: septic shock is not a single pathophysiological state. Patients who share the same diagnostic label may have different combinations of vasoplegia, myocardial dysfunction, endothelial injury, glycocalyx degradation, capillary leak, microcirculatory failure, mitochondrial dysfunction, hyperinflammation, and sepsis-induced immunosuppression. This heterogeneity affects prognosis and treatment response. It also explains why interventions supported by strong biological rationale may produce neutral results when applied to broad, unselected populations (7–10).

The Surviving Sepsis Campaign has identified personalized and precision-medicine approaches as research priorities, particularly for determining which interventions benefit which patients and at which time points (11). Recent evidence comparing restrictive and liberal fluid strategies has not shown a universal mortality advantage for either approach, reinforcing the need to tailor fluid intensity to fluid responsiveness, fluid tolerance, and perfusion trajectory (12). Similarly, systematic reviews of early norepinephrine suggest potential benefits in selected patients through faster hemodynamic stabilization and lower fluid exposure, while emphasizing the need for better patient selection (13). These observations support a move from fixed escalation toward physiology-guided resuscitation.

Existing syntheses have generally concentrated on one layer of the precision-sepsis problem, including sepsis subphenotypes and theragnostics (9), the timing of norepinephrine initiation (13), host-response modulation (14), or the broader immunotherapy landscape (15). The novelty of the present review is therefore not the proposal of a new phenotype, endotype, or biomarker. Rather, it is the clinically oriented integration of three therapeutic domains—fluid therapy, vasopressor support, and immunomodulation—with two cross-cutting precision layers: biological phenotyping and bedside monitoring. This structure links classification to immediate treatment choices, stopping rules, repeated reassessment, and de-escalation while preserving the priority of time-critical guideline-based care.

## 2 Evidence search and selection

This narrative review was informed by targeted searches of PubMed/MEDLINE and the Cochrane Library from database inception to 4 August 2026. Searches were supplemented by backward screening of the reference lists of eligible guidelines, trials, and reviews and by forward citation tracking of key studies. Search concepts combined “sepsis” or “septic shock” with terms related to precision medicine and heterogeneity (“precision medicine,” “personalized medicine,” “phenotype,” “subphenotype,” and “endotype”); fluid therapy (“fluid resuscitation,” “fluid responsiveness,” “fluid tolerance,” “balanced crystalloids,” “albumin,” and “de-resuscitation”); vasopressor therapy (“norepinephrine,” “vasopressin,” “angiotensin II,” and “renin”); immunomodulation (“immunoparalysis,” “mHLA-DR,” “anakinra,” “interferon-gamma,” and “interleukin-7”); and endothelial or bedside assessment (“endothelial dysfunction,” “glycocalyx,” “microcirculation,” “capillary refill time,” “point-of-care ultrasound,” and “venous congestion”). The final search update was completed on 4 August 2026.

Sources were selected purposively according to clinical relevance and evidentiary strength. Priority was given to current international guidelines, consensus definitions, adult randomized clinical trials, individual-patient or prespecified secondary analyses, systematic reviews, meta-analyses, and mechanistic or biomarker studies that directly informed treatment selection or biological stratification. When a guideline or narrative review supported a therapeutic recommendation, the principal primary studies were also sought. Older landmark studies were retained when they remained foundational, whereas superseded recommendations were updated using current evidence. Adult sepsis and septic shock evidence was prioritized; selected pediatric or translational studies were included only when they provided unique phenotyping or mechanistic insight not available from adult cohorts. English-language full-text publications were considered. Because this was a narrative rather than systematic review, no protocol was registered, no formal risk-of-bias tool was applied, and the search was not intended to be exhaustive.

## 3 Septic shock as a heterogeneous syndrome: clinical phenotypes and biological endotypes

At the bedside, septic shock is recognized as infection-associated circulatory failure with vasopressor requirement and impaired tissue perfusion. This operational definition is essential for urgent treatment, but it compresses diverse biology into one label. Patients may differ in age, comorbidity, pathogen, infection source, time from onset, prior antimicrobial exposure, cardiac reserve, renal function, endothelial integrity, baseline immune competence, and exposure to corticosteroids, chemotherapy, or other immunosuppressive therapies (16). These differences are not merely descriptive; they influence shock trajectory, organ failure, fluid responsiveness, vasopressor dose, susceptibility to secondary infection, and likelihood of benefit or harm from adjunctive therapy.

Clinical heterogeneity is often visible early. Some patients present with profound vasoplegia and warm extremities despite preserved or high cardiac output. Others have septic myocardial dysfunction, right ventricular dysfunction, low-output physiology, or mixed shock (17). Lactate trajectories may also be discordant with systemic hemodynamics; their mechanistic interpretation is addressed in Section 8 (18, 19). Infection source also matters: necrotizing soft-tissue infections may deteriorate rapidly when source control is delayed, and surgical treatment within early time windows is associated with lower mortality (20). Thus, the same diagnosis may require different dominant priorities: source control, cautious fluid administration, early vasopressors, echocardiography-guided inotropy, ventilatory optimization, renal support, or immune-risk stratification.

Biological heterogeneity is less visible but equally important. Sepsis may involve simultaneous or sequential hyperinflammation and immunosuppression; immune suppression may coexist with ongoing inflammatory injury (14, 21). Endothelial dysfunction and glycocalyx injury contribute to capillary leak, tissue edema, microvascular thrombosis, leukocyte adhesion, impaired oxygen delivery, and fluid intolerance (22–25). Transcriptomic and biomarker studies support the existence of sepsis endotypes with divergent immune, inflammatory, metabolic, and prognostic profiles (10, 26, 27).

A useful precision framework therefore distinguishes clinical phenotypes from biological endotypes. Clinical phenotypes are observable bedside patterns derived from vital signs, organ dysfunction, echocardiography, ultrasound, perfusion assessment, and treatment response. Biological endotypes refer to underlying mechanisms, such as hyperinflammatory, immunoparalysed, endothelial-injury-dominant, interferon-driven, immunometabolic, or adaptive immune-suppression states. The two do not necessarily align: patients with similar MAP values, vasopressor requirements, or bedside phenotypes may have different immune, endothelial, or metabolic drivers. Precision septic shock care therefore requires integration of rapid bedside phenotyping with repeated reassessment and, where feasible, biomarker refinement (26–30).

Several published classification systems illustrate why phenotype and endotype should not be treated as interchangeable. The SENECA analysis identified four clinical phenotypes (alpha, beta, gamma, and delta) from routinely available data; the delta phenotype was enriched for septic shock, hepatic dysfunction, and high mortality (28). In contrast, transcriptomic studies identified two sepsis response signatures (SRS1 and SRS2), with SRS1 showing relative immunosuppression, endotoxin tolerance, T-cell exhaustion, and higher early mortality (29), while the MARS programme identified four molecular endotypes, of which MARS1 showed prominent immune suppression and the poorest outcome profile (26). A direct comparison in 522 critically ill patients found limited patient-level concordance among clinical, plasma-biomarker, MARS, and SRS classifications despite partial overlap in biological pathways (27). These schemes therefore capture complementary layers of heterogeneity rather than a single validated taxonomy, and none is currently established as a routine bedside treatment classifier.

Figure 1 summarizes the relationship between observable clinical phenotypes and underlying biological endotypes, while Table 1 links representative phenotypes and endotypes to their principal bedside clues and therapeutic priorities.

**Figure 1:**
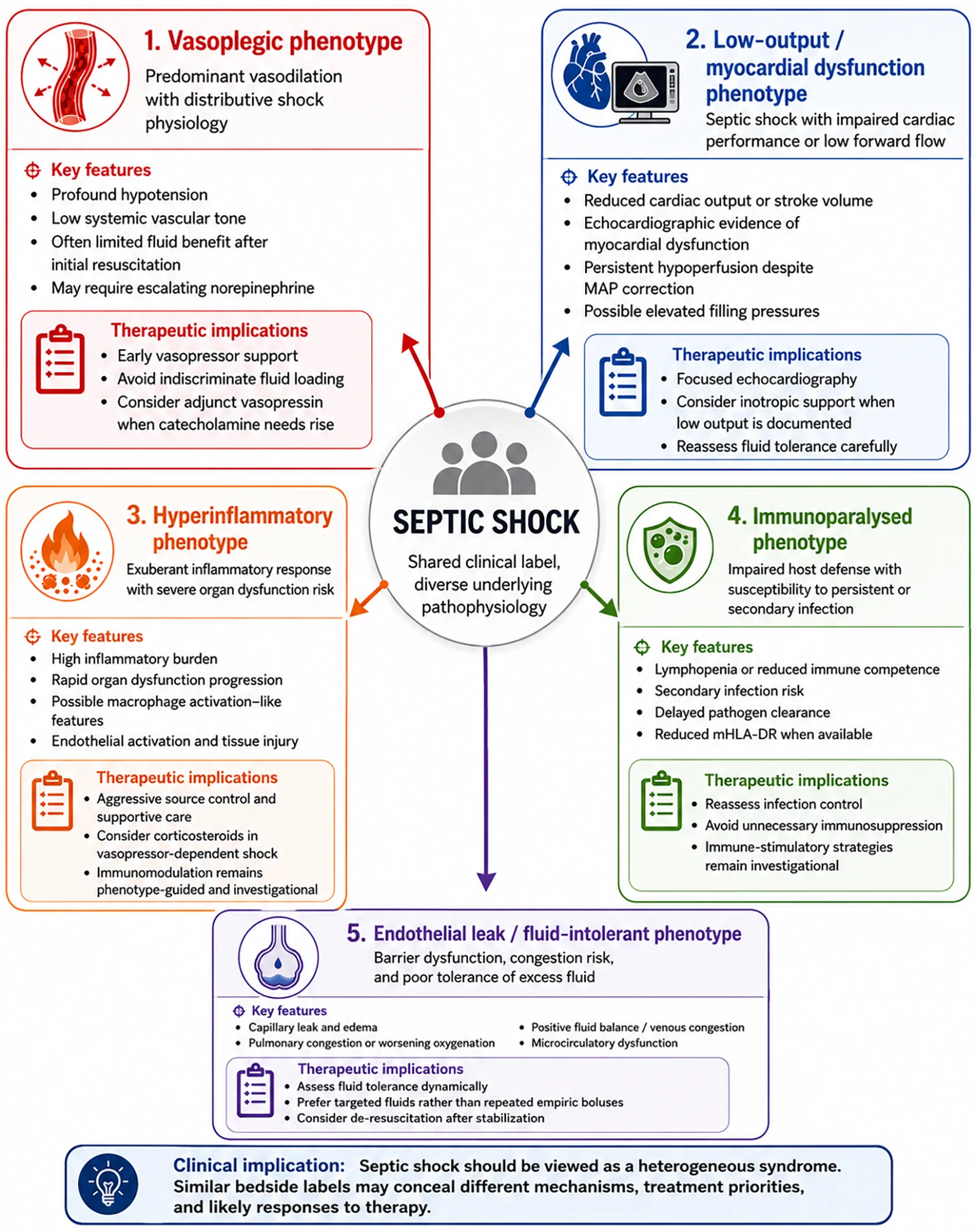
Heterogeneity of septic shock: One syndrome, distinct clinical phenotypes and biological endotypes. Conceptual overview of the clinical and biological heterogeneity underlying septic shock. Although patients may meet the same diagnostic criteria, their dominant pathophysiological states can differ substantially. Clinical phenotypes include vasoplegic shock, low-output or myocardial dysfunction, and endothelial leak or fluid-intolerant states. Biological endotypes include hyperinflammatory, immunoparalysed, endothelial-injury-dominant, immunothrombotic, and immunometabolic states. These categories may overlap and evolve over time. The figure emphasizes that precision care requires moving beyond a single syndromic label toward identification of the dominant mechanism driving shock at a given time point. MAP, mean arterial pressure; mHLA-DR, monocyte human leukocyte antigen-DR.

**Table 1:** Clinical phenotype- and biological endotype-directed therapy matrix in septic shock.

Dominant phenotype or endotype	Representative bedside clues	Physiological interpretation	Core therapeutic priorities
Vasoplegic phenotype	Profound hypotension, warm extremities, wide pulse pressure, escalating vasopressor requirement, limited benefit from repeated fluids.	Normal or high cardiac output with low vascular tone; MAP below target while preload responsiveness may be limited.	Early norepinephrine with dynamic perfusion reassessment and avoidance of non-beneficial fluids; add vasopressin when norepinephrine requirements escalate, and reserve angiotensin II for selected refractory vasodilatory shock.
Low-output/myocardial dysfunction phenotype	Cool extremities, weak pulses, narrow pulse pressure, persistent hypoperfusion despite MAP correction, echocardiographic dysfunction.	Reduced stroke volume or cardiac output; congestion may coexist with hypoperfusion.	Focused echocardiography, avoid indiscriminate fluids if congestion is present, consider inotropy when low output and hypoperfusion are documented.
Hyperinflammatory biological endotype	Rapid deterioration, high fever, early multiorgan dysfunction, possible macrophage-activation-like features.	Often vasoplegic or mixed, with capillary leak and early fluid intolerance.	Urgent source control and supportive care; avoid nonspecific immunosuppression without evidence of hyperinflammatory biology; consider trial-based or protocolized immune-directed therapy only when endotype criteria are met.
Immunoparalysed biological endotype	Recurrent or secondary infection, impaired pathogen clearance, persistent critical illness, lymphopenia.	No single hemodynamic pattern; may coexist with late vasoplegia or organ dysfunction.	Reassess infection control, avoid unnecessary immunosuppression, monitor for secondary infection, and consider trial-based immune-stimulatory strategies only when immunoparalysis criteria are met.
Endothelial leak/fluid-intolerant phenotype	Rapid edema after modest fluids, worsening oxygenation with boluses, positive fluid balance, venous congestion.	Transient or absent fluid responsiveness with poor tolerance; pulmonary or systemic congestion becomes dominant.	Stop non-beneficial fluids, use vasopressors when appropriate, assess congestion, and consider deresuscitation after stabilization. No endothelial-targeted drug is established for routine care; biomarker-guided use should remain within clinical trials.
Mixed/transitional phenotype	Features of more than one phenotype; discordance between MAP, lactate, capillary refill, organ dysfunction, and biomarker classification; some patients remain unclassified by discrete immune thresholds.	Mechanisms change over hours to days; clinical and biological classifiers may capture different layers and may not assign the same patient consistently.	Repeat phenotyping, treat the current dominant mechanism, avoid forcing uncertain patients into a binary immune category, and consider advanced stratification or trial eligibility when available.

The therapeutic implication is straightforward: the question should shift from “Does the patient have septic shock?” to “What is the dominant mechanism of shock now, and what intervention is most likely to help without causing avoidable harm?” This dynamic therapeutic matching provides the basis for precision fluid therapy, precision vasopressor use, and targeted immunomodulation.

## 4 Precision fluid therapy

### 4.1 Reframing intravenous fluids as a titrated therapy

Intravenous fluids remain central to early septic shock resuscitation, especially when hypoperfusion reflects relative hypovolemia or preload reserve. The 2026 Surviving Sepsis Campaign suggests administering at least 30 mL/kg of intravenous crystalloid within the first 3 hours to adults with sepsis-induced hypoperfusion or septic shock, but the certainty of evidence is low (6). In a precision framework, this recommendation is best understood as an initial population-level default rather than a mandate for unexamined, uninterrupted fluid delivery. The urgency and total amount of initial resuscitation should be balanced against comorbidity, the severity of hypoperfusion, evolving vasopressor requirements, and repeated assessment of whether each clinically meaningful increment is improving perfusion without causing congestion. Current guidance also states that, after 30 mL/kg has been administered and hypoperfusion persists, either a liberal or restrictive strategy may be selected according to individual patient and health-system factors (6). Fluids are not benign: once preload reserve is exhausted, additional volume may increase venous pressure, tissue edema, pulmonary congestion, renal venous congestion, and abdominal compartment physiology without improving forward flow (31, 32). During sepsis, endothelial injury and glycocalyx degradation reduce intravascular fluid persistence and promote interstitial accumulation, making some patients fluid intolerant early in the course (22).

Precision fluid therapy begins with fluid responsiveness: the likelihood that preload augmentation will increase stroke volume or cardiac output. Dynamic assessment is more informative than static filling pressures because it tests cardiovascular response. Passive leg raising, mini-fluid challenge, stroke-volume variation, pulse-pressure variation, end-expiratory occlusion testing, and echocardiographic measures such as LVOT-VTI can help determine whether a bolus is likely to improve forward flow (33–35). In the FRESH trial, passive-leg-raise-guided resuscitation in sepsis-associated hypotension and shock reduced 72-hour net fluid balance and was associated with lower rates of renal replacement therapy and mechanical ventilation compared with usual care (36). A 2025 systematic review of nine randomized trials involving 698 patients found that dynamic-guided resuscitation probably reduced 28-day mortality (moderate-certainty evidence) and may reduce acute kidney injury and day-3 cumulative fluid balance (low-certainty evidence), while effects on ICU mortality and several organ-support outcomes remained uncertain (37).

Fluid responsiveness alone is not enough. A patient may be fluid responsive yet unable to tolerate additional fluid without clinically important harm. Fluid tolerance has been defined as the degree to which a patient can receive fluid without developing organ dysfunction and was proposed to complement, rather than replace, assessment of fluid responsiveness (38). Practical assessment should integrate oxygenation, lung ultrasound B-lines, echocardiographic ventricular function, venous Doppler congestion patterns where available, cumulative fluid balance, capillary refill time, urine output, lactate trajectory, vasopressor dose, and edema. This construct remains clinically useful but is not yet a fully validated treatment algorithm; therefore, its signals should be interpreted sequentially and in context. Persistent lactate should prompt integrated reassessment rather than reflexive boluses when preload responsiveness is absent or fluid intolerance is present (see Section 8) (18, 38, 39).

Figure 2 illustrates the four possible combinations of fluid responsiveness and fluid tolerance and their implications for cautious fluid administration.

**Figure 2:**
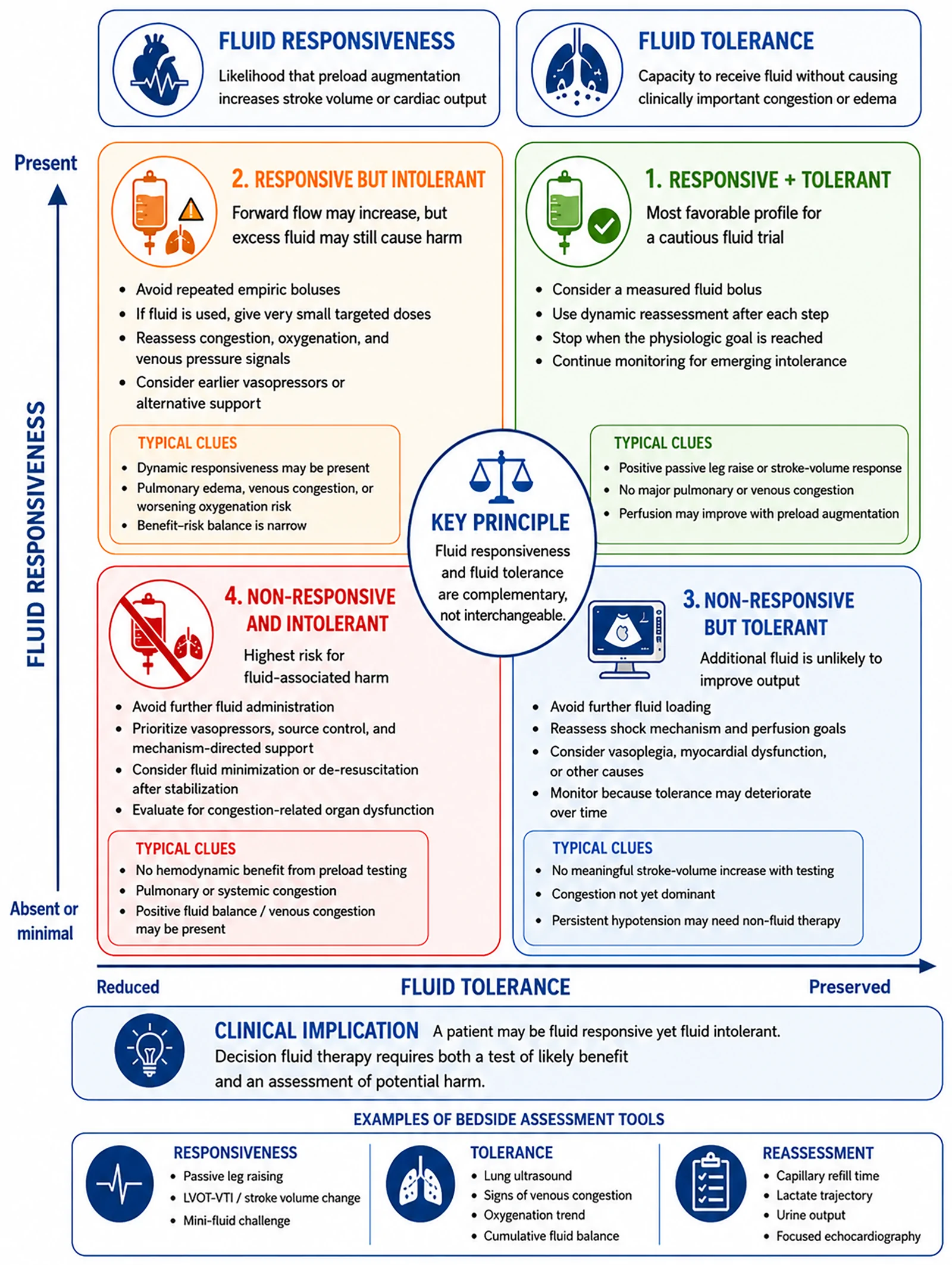
Fluid responsiveness and fluid tolerance in septic shock. Precision fluid therapy in septic shock requires simultaneous evaluation of two complementary but distinct physiological properties: fluid responsiveness and fluid tolerance. Fluid responsiveness refers to the likelihood that preload augmentation will increase stroke volume or cardiac output, whereas fluid tolerance reflects the capacity to receive additional intravenous fluid without clinically important congestion, edema, or worsening organ dysfunction. The four-quadrant framework illustrates that patients may be fluid responsive yet intolerant, non-responsive but still tolerant, responsive and tolerant, or neither responsive nor tolerant. The most favorable profile for a cautious fluid trial is the responsive-and-tolerant quadrant. Conversely, patients who are non-responsive and intolerant are at greatest risk for fluid-associated harm. This framework supports dynamic reassessment rather than repeated empiric fluid boluses. LVOT-VTI, left ventricular outflow tract velocity–time integral.

Restrictive-fluid trials should not be interpreted as proof that every patient should receive less fluid from the outset, nor should neutral superiority trials be described as establishing safety or equivalence. In CLOVERS, patients with sepsis-induced hypotension who had already received 1–3 L of crystalloid were assigned to a restrictive strategy prioritizing vasopressors or a liberal fluid strategy; death before discharge home by day 90 occurred in 14.0% and 14.9%, respectively, with no significant difference (12). CLASSIC enrolled ICU patients with septic shock after initial resuscitation and found similar 90-day mortality with restrictive and standard fluid therapy (42.3% vs 42.1%) (40). In ARISE-FLUIDS, adults presenting to the emergency department with septic shock had the same median number of days alive and out of hospital through day 90 with restricted fluids and earlier vasopressors versus greater fluid volumes and later vasopressors (76 days in both groups; difference, 0.0 days). The vasopressor-forward group received a median of 1108 mL less fluid during the first 24 hours, and pulmonary edema occurred in 0.6% versus 5.0%, but the primary patient-centered outcome was neutral (41). Collectively, these trials demonstrate that materially different fluid-vasopressor strategies can produce similar primary outcomes in broad populations; they do not establish a universally superior strategy. The precision implication is to provide timely initial crystalloid when hypoperfusion is present, stop non-beneficial fluid early, and individualize subsequent fluid exposure according to responsiveness, tolerance, perfusion trajectory, and congestion (6, 12, 40, 41).

Deresuscitation should be considered after shock stabilization when perfusion goals are achieved or improving and signs of fluid accumulation are clinically meaningful. Strategies include discontinuing unnecessary maintenance fluids, minimizing hidden fluid from drug diluents, concentrating infusions, using diuretics when appropriate, and applying ultrafiltration in selected patients. Current evidence supports physiological plausibility and potential improvements in ventilator-free days or ICU length of stay, but mortality benefit remains uncertain and patient selection is critical (42, 43).

A practical precision-fluid cycle includes five questions: Is there tissue hypoperfusion that may improve with circulatory intervention? Is the patient preload responsive? Can the patient tolerate fluid? Did the bolus achieve a predefined endpoint? Has the patient entered a stabilization or deresuscitation phase? Each bolus should have an indication, dose, endpoint, and stopping rule. Precision fluid therapy means giving the right amount of fluid to the right patient at the right time, not simply giving less fluid.

### 4.2 Selection of resuscitation fluid

Precision fluid therapy concerns not only the volume and timing of administration but also the composition of the infused solution. Current international guidelines recommend crystalloids as first-line resuscitation fluid and suggest balanced crystalloids over 0.9% saline during initial resuscitation, while favoring saline in traumatic brain injury (6). The primary trial evidence is directionally favorable but not uniform. In SMART, balanced crystalloids modestly reduced the composite of death, new renal-replacement therapy, or persistent renal dysfunction at 30 days compared with saline (14.3% vs 15.4%) (44). A secondary analysis of SMART participants with sepsis found lower 30-day in-hospital mortality with balanced crystalloids (26.3% vs 31.2%), but this was a subgroup analysis rather than a dedicated sepsis trial (45). By contrast, the multicenter BaSICS trial found no significant reduction in 90-day mortality with a balanced solution compared with saline (26.4% vs 27.2%) (46). These findings support a preference for balanced crystalloids in most patients while acknowledging that the absolute effect is likely small and heterogeneous. Balanced solutions reduce chloride exposure and hyperchloremic acidosis, but clinical benefit may depend on baseline renal and neurological status, total fluid volume, and the specific solution used (6, 44–46).

Routine colloid use is not supported. The 2026 guideline suggests crystalloids alone rather than crystalloids with routine supplemental albumin, while allowing selective albumin use after large crystalloid requirements or in cirrhosis and advising against albumin in traumatic brain injury (6). In ALBIOS, adding albumin to crystalloids did not improve 28- or 90-day survival in patients with severe sepsis or septic shock (47). More recently, the prematurely terminated ARISS trial found no significant improvement in 90-day mortality with albumin replacement in septic shock (43.3% vs 45.9%), leaving residual uncertainty but no basis for routine replacement (48, 49). Hydroxyethyl starch should not be used: in the sepsis-specific 6S trial, HES 130/0.42 increased 90-day mortality and the need for renal-replacement therapy compared with Ringer's acetate (50). Gelatin solutions are also discouraged by current guidelines because evidence of benefit is lacking and safety uncertainty remains (6). Fluid composition should therefore be selected according to neurological status, renal and hepatic function, cumulative crystalloid exposure, cost, and the anticipated balance between intravascular benefit and interstitial accumulation.

## 5 Precision vasopressor therapy

Vasopressors are essential in septic shock because persistent hypotension often reflects vasodilation, vascular hyporeactivity, altered autonomic regulation, endothelial dysfunction, and maldistribution of blood flow (6, 51). Restoring mean arterial pressure (MAP) is necessary, but it does not guarantee restoration of microcirculatory flow, tissue oxygen extraction, renal perfusion, or cellular oxygen utilization (52, 53). Precision vasopressor therapy should therefore match timing, dose, agent selection, perfusion targets, and weaning to the dominant circulatory phenotype.

The 2026 Surviving Sepsis Campaign recommends norepinephrine as the first-line vasopressor over dopamine, epinephrine, or selepressin in adults with septic shock (6). The landmark SOAP II randomized trial enrolled patients with shock of mixed etiologies rather than septic shock exclusively; it found no significant difference in 28-day mortality between dopamine and norepinephrine (52.5% vs. 48.5%; *P* = 0.10), but dopamine caused substantially more arrhythmic events (24.1% vs. 12.4%; *P* < 0.001) (54). These data support norepinephrine's preferred safety profile while also illustrating that some foundational comparative evidence is not sepsis-specific. The precision question is therefore not whether norepinephrine is first line, but when to start it and when to add a mechanistically distinct agent.

In vasoplegic shock, delaying vasopressors while repeatedly administering fluid may prolong hypotension and increase fluid accumulation. The phase II CENSER randomized trial directly tested early low-dose norepinephrine in 310 adults with sepsis and hypotension. Shock control by 6 hours was achieved more often with early norepinephrine than with standard care (76.1% vs. 48.4%; *P* < 0.001), whereas 28-day mortality did not differ significantly (15.5% vs. 21.9%; *P* = 0.15) (55). Updated meta-analyses similarly suggest that earlier norepinephrine may reduce early fluid exposure and shorten time to MAP restoration, but the certainty of any mortality benefit remains limited (13, 56). Early vasopressors should not replace fluid assessment. They are most logical when hypotension is severe, preload responsiveness is absent or limited, capillary leak is prominent, or fluid intolerance is likely; patients with demonstrable fluid-responsive hypovolemia may still benefit from cautious fluid expansion.

Agent selection should also be mechanistic. Septic vasodilation reflects multiple pathways, including adrenergic dysregulation, relative vasopressin deficiency, nitric oxide-mediated vasodilation, altered vascular smooth-muscle responsiveness, and renin-angiotensin-system dysregulation (51, 57). Current guidance suggests adding vasopressin when norepinephrine requirements are escalating rather than replacing norepinephrine as the routine initial agent (6). An individual-patient-data meta-analysis found no clear 28-day mortality reduction with vasopressin-based strategies, but reported fewer arrhythmias and more digital ischemia (58). VANISH tested early vasopressin as an alternative first-line vasopressor, not as an adjunct: its two-part primary kidney-failure-free-day outcome was not improved compared with norepinephrine; among patients who died, developed kidney failure, or both, the median was 9 versus 13 days (difference, −4 days; 95% CI, −11 to 5) (59). Accordingly, VANISH does not directly establish the benefit of adjunctive vasopressin. Angiotensin II is a mechanistically distinct option for selected refractory vasodilatory shock; biomarker analyses suggest that elevated renin may identify patients more likely to respond, although validated selection criteria remain investigational (60, 61).

An emerging extension of this approach is multimodal vasopressor therapy, in which agents acting through complementary pathways are used to avoid extreme catecholamine escalation. Norepinephrine primarily restores vascular tone through adrenergic mechanisms, vasopressin acts through V1 receptors, and angiotensin II targets the renin-angiotensin system. In a 2024 meta-analysis of 51 randomized trials, non-adrenergic vasopressors were associated with lower mortality in the septic-shock subgroup (43% vs. 48%; risk ratio 0.92, 95% CI 0.86–0.97), as well as lower pooled risks of renal replacement therapy and atrial fibrillation; however, these secondary outcomes were based on smaller subsets of trials, and no individual non-adrenergic agent showed a statistically significant survival benefit (62). The analysis therefore supports the biological plausibility of catecholamine-sparing therapy but does not prove that routine simultaneous early administration of multiple vasopressors improves outcomes. Multimodal therapy should currently remain a selected, phenotype-directed strategy for persistent vasoplegia or rapidly escalating norepinephrine requirements.

Renin is also emerging as a marker of renin–angiotensin–aldosterone system dysregulation in circulatory shock. Elevated or persistently increasing renin may reflect reduced renal perfusion, diminished endogenous angiotensin II activity, impaired conversion of angiotensin peptides, or severe vasodilatory stress (51, 63). Serial renin kinetics have demonstrated prognostic value, and some studies suggest that changes in renin may outperform lactate kinetics in predicting mortality among hypotensive critically ill patients (64). However, septic-shock-specific evidence is not entirely uniform, with other studies reporting mortality discrimination comparable to lactate but better prediction of acute kidney injury (65). Renin may also help identify patients more likely to respond to angiotensin II (60). Nevertheless, assay availability, measurement timing, treatment effects, and the absence of validated therapeutic thresholds currently limit its use as a routine resuscitation target (63).

MAP targets must be interpreted as an initial reference point, not a complete definition of perfusion. The 2026 Surviving Sepsis Campaign recommends an initial MAP target of approximately 65 mmHg, with subsequent adjustment according to clinical context (6). In SEPSISPAM, targeting 80–85 mmHg rather than 65–70 mmHg did not reduce overall mortality; patients with chronic hypertension required less renal replacement therapy, but higher targets increased atrial fibrillation (66). OPTPRESS directly evaluated this question in adults aged 65 years or older: its primary outcome, 90-day all-cause mortality, was higher with a MAP target of 80–85 mmHg than with 65–70 mmHg (39.3% vs. 28.6%; risk difference 10.7%, 95% CI 2.6–18.9) (67). The trial was terminated early after the interim analysis met the prespecified stopping criterion for ineffectiveness and suggested harm from the high-MAP strategy. Because trials stopped early for apparent benefit or harm can yield exaggerated effect estimates, the magnitude of the observed mortality difference should be interpreted cautiously. Importantly, no clinical benefit of the high-MAP strategy was observed for any outcome in any examined subpopulation, including patients with known chronic hypertension, contrasting with the renal-replacement signal observed in the chronic-hypertension subgroup of SEPSISPAM (67). Taken together, these findings argue against routine high MAP targets in older patients while preserving the need for individualized adjustment when organ perfusion remains inadequate.

Tissue perfusion should therefore be assessed alongside MAP. Capillary refill time, skin mottling, urine output, mental status when assessable, lactate kinetics, echocardiographic cardiac output estimates, venous congestion, and signs of ischemia should influence vasopressor titration (18, 52, 53, 63, 68). A patient whose MAP increases with norepinephrine and whose perfusion improves differs from a patient whose MAP normalizes while lactate rises, urine output falls, or extremities become ischemic. Precision vasopressor therapy requires attention to this relationship between arterial pressure and tissue perfusion.

Precision also applies to vasopressor weaning. Prolonged exposure may contribute to arrhythmia, excessive vasoconstriction, myocardial stress, peripheral ischemia, or impaired regional perfusion, whereas premature discontinuation may cause recurrent hypotension and renewed fluid loading. Weaning should be attempted when source control is adequate, perfusion markers are stable or improving, lactate is clearing or explained by non-hypoperfusion mechanisms, organ function is acceptable, and fluid status is optimized. The optimal sequence for discontinuing norepinephrine and vasopressin remains uncertain, so de-escalation should be individualized according to dose, trajectory, adverse effects, and institutional practice (69, 70).

## 6 Biological endotyping: immune, endothelial, and coagulation axes

Hemodynamics alone cannot define septic shock biology. Shock reflects not only arterial pressure and circulating volume but also host-response dysregulation, endothelial injury, microvascular failure, coagulation activation, and metabolic dysfunction. Immune and endothelial phenotyping should be viewed as complementary to clinical judgment: tools that may help explain why patients with similar MAP, lactate, or vasopressor dose have different trajectories and treatment responses (14, 15, 22, 30, 71).

Sepsis immune dysregulation is multidimensional and dynamic. Hyperinflammation and immunoparalysis may occur sequentially, coexist in the same patient, or differ across circulating and tissue compartments (14, 21, 30, 72, 73). Hyperinflammatory states may involve excessive innate immune activation, cytokine amplification, endothelial and complement activation, coagulation disturbance, and tissue injury. Immunoparalysis may involve impaired antigen presentation, reduced monocyte HLA-DR expression, lymphocyte depletion or dysfunction, impaired *ex vivo* cytokine production, inhibitory-checkpoint expression, and susceptibility to secondary infection. A 2025 consensus analysis integrating more than 7,000 samples across 37 cohorts further supported myeloid and lymphoid dysregulation as continuous, overlapping axes associated with severity and mortality rather than a simple binary sequence (30). These states have different therapeutic implications: suppressing inflammation in a profoundly immunoparalysed patient may be harmful, while immune stimulation during uncontrolled hyperinflammation may worsen tissue injury.

Candidate immune biomarkers include monocyte HLA-DR, lymphocyte counts and subsets, cytokines such as IL-6 and IL-10, soluble immune checkpoints, neutrophil function, complement activation markers, ferritin, and transcriptomic signatures (15, 21, 30, 71, 72). Transcriptomic analyses provide an important proof of concept for predictive enrichment. Davenport et al. derived SRS1 and SRS2 in adults with sepsis due to community-acquired pneumonia, with SRS1 associated with relative immunosuppression and higher early mortality (29). In a secondary analysis of the VANISH randomized trial, corticosteroid treatment showed a differential association with outcome by SRS endotype; after a subsequent correction of endotype assignments, hydrocortisone remained associated with increased mortality in SRS2 (odds ratio, 4.6; 95% CI, 1.5–14.4) (74, 75). Because this was an exploratory secondary analysis, randomization was not stratified by endotype, and only a subset had transcriptomic data, the finding is hypothesis-generating rather than a basis for withholding corticosteroids in current practice. Monocyte HLA-DR and ferritin have also been used prospectively for trial enrichment (76, 77), but routine implementation requires assay harmonization, rapid turnaround, validated thresholds, and evidence that test-guided treatment improves patient-centered outcomes.

The endothelium is a central organ of septic shock. It regulates barrier function, vascular tone, leukocyte trafficking, coagulation, platelet interaction, and microcirculatory flow. Glycocalyx injury and endothelial activation promote capillary leak, tissue edema, microvascular thrombosis, impaired oxygen diffusion, fluid intolerance, and organ dysfunction (22–24). Syndecan-1 and endocan have prognostic associations in sepsis (23–25), whereas angiopoietin-pathway markers remain investigational for biological stratification and therapeutic enrichment (78). No endothelial biomarker has a validated treatment threshold for routine septic shock care.

Septic capillary leakage is a dynamic manifestation of endothelial barrier failure and may improve as infection is controlled and endothelial junctional and glycocalyx integrity recover. However, biological reversibility should not be conflated with a proven drug effect: no pharmacological treatment has been shown to directly and reliably reverse capillary leakage and improve patient-centered outcomes in routine septic shock care. Current management therefore remains causal and supportive, including rapid antimicrobial therapy and source control, avoidance of unnecessary hydrostatic and fluid burden, and transition to fluid minimization or active fluid removal after the resuscitation phase when clinically appropriate (42, 43, 78).

Evidence for endothelial-targeted interventions spans markedly different translational stages and should not be presented as equivalent. In murine polymicrobial sepsis, the synthetic Tie2 agonist peptide vasculotide reduced vascular leakage and improved survival (79). A separate preclinical study showed that an angiopoietin-2-binding and Tie2-activating antibody (ABTAA) strengthened endothelial and glycocalyx integrity, reduced vascular leakage and organ injury, and improved survival in murine sepsis models (80). These findings support the biological plausibility of the angiopoietin-Tie2 axis, but no Tie2-directed therapy has yet demonstrated clinical efficacy in septic shock.

The adrenomedullin pathway has progressed to biomarker-guided human testing. AdrenOSS-2 randomized 301 adults with early septic shock and elevated bioactive adrenomedullin to a single infusion of adrecizumab or placebo. The phase 2a trial established acceptable safety and tolerability, but it was not powered for definitive efficacy; exploratory outcomes did not establish statistically significant improvements in 28-day mortality, vasopressor duration, or fluid balance (81). The trial therefore supports the feasibility of endothelial biomarker enrichment, not routine adrecizumab use.

Other strategies remain earlier in development. In a lipopolysaccharide-induced animal model, doxycycline pretreatment attenuated glycocalyx shedding, vascular hyperpermeability, neutrophil transmigration, and microvascular impairment (82). By contrast, the first-in-human Cellular Immunotherapy for Septic Shock study administered allogeneic mesenchymal stromal cells to nine patients in a phase 1 dose-escalation trial designed primarily to assess safety and biological effects, not clinical efficacy (83). These data do not justify routine glycocalyx-directed or cell-based therapy.

Accordingly, endothelial-targeted interventions should be described by their evidence level: Tie2 and glycocalyx-preserving approaches are predominantly preclinical; adrecizumab has phase 2a biomarker-guided safety data without definitive outcome benefit; and mesenchymal stromal cells have early-phase feasibility data. Endothelial biomarkers may eventually support enrichment of a capillary-leak-dominant endotype, but validated treatment thresholds and reproducible outcome benefits remain absent.

Coagulation endotyping is also relevant because inflammation and thrombosis are tightly linked in septic shock. Immunothrombosis, platelet activation, endothelial injury, and microvascular clot formation may contribute to organ dysfunction even when systemic hemodynamics appear corrected. At present, routine coagulation tests are mainly used for risk assessment and supportive decisions rather than precision therapy. Neutral or inconsistent results from anticoagulant and endothelial-coagulation trials in broad sepsis populations, including the SCARLET thrombomodulin trial, reinforce the need for predictive enrichment rather than unselected application (84). Future biomarker-guided strategies may identify subgroups more likely to benefit from anticoagulant, endothelial-targeted, or immune-thrombotic interventions (15, 71).

The key practical point is that phenotyping must be dynamic. A patient classified as hyperinflammatory, immunoparalysed, or fluid intolerant at admission may transition over hours to days, and a substantial proportion of patients may not fit a prespecified discrete category. Repeated reassessment is therefore essential, especially for immunomodulatory therapy, because treatment direction depends on timing, source control, immune trajectory, organ dysfunction, and the validity of the classifier used (27, 30, 77).

## 7 Immunomodulation: promise, caution, and precision

Immunomodulation is one of the most attractive but difficult frontiers in septic shock. Because sepsis is defined by a dysregulated host response to infection, targeting that response is biologically rational (5). However, decades of trials evaluating anti-inflammatory, immune-stimulatory, antibody-based, anticoagulant, complement-directed, and cytokine-targeted interventions have produced inconsistent results in broad, biologically unselected populations (14, 15, 30). The central problem is not simply whether an intervention is biologically active, but whether the relevant immune abnormality is present, whether it persists long enough to be measured, and whether the selected biomarker predicts treatment response rather than prognosis alone.

Corticosteroids have the most established clinical role among immune-active adjuncts. The 2026 Surviving Sepsis Campaign suggests intravenous corticosteroids for adults with septic shock, while the regimen most commonly represented in trials is hydrocortisone 200 mg/day (6). Large randomized trials differed in their survival findings: ADRENAL found no significant reduction in 90-day mortality but faster shock resolution, whereas APROCCHSS reported lower 90-day mortality with hydrocortisone plus fludrocortisone (85, 86). A 2024 systematic review of 45 randomized trials involving 9,563 patients found that corticosteroids probably reduce short-term mortality and increase shock reversal, while probably increasing hyperglycemia and hypernatremia and possibly increasing neuromuscular weakness (87). Transcriptomic treatment-effect analyses from VANISH suggest that corticosteroid response may differ by immune endotype (74, 75), but these exploratory data do not supersede current guideline-based treatment because prospective endotype-stratified confirmation is lacking.

Anti-inflammatory strategies may be most appropriate for biologically selected hyperinflammatory states. Interleukin-1 receptor blockade with anakinra is a leading example: reanalysis of a prior phase 3 trial suggested a survival signal in patients with macrophage-activation-like features (88), and the PROVIDE trial demonstrated the feasibility of assigning immunotherapy according to prespecified immune dysfunction (76). These findings support enrichment strategies but do not justify empiric anakinra use in unselected septic shock.

ImmunoSep provides the clearest prospective test of this principle. The phase 2b trial enrolled adults with Sepsis-3 caused by pneumonia or primary bacteremia who met laboratory criteria for either macrophage activation-like syndrome (ferritin >4420 ng/mL) or sepsis-induced immunoparalysis (ferritin <=4420 ng/mL and fewer than 5000 HLA-DR receptors per CD45/CD14 monocyte); the population was not restricted to septic shock (77). Of 672 patients screened, 281 (41.8%) were randomized and 276 were included in the primary analysis; 355 screened patients (52.8%) had an unclassified immune state and were not enrolled. The primary endpoint, a decrease of at least 1.4 points in mean SOFA score by day 9, occurred in 35.1% with precision immunotherapy versus 17.9% with placebo (*P* = .002). Twenty-eight-day mortality was 43.5% versus 49.7% (*P* = .34), with no significant difference in 90-day mortality. Serious treatment-emergent adverse events were common in both groups; anemia was more frequent in the anakinra subgroup and hemorrhage in the recombinant interferon-gamma subgroup (77). ImmunoSep therefore supports feasibility and an organ-dysfunction signal, but not a proven mortality benefit or routine use, and it highlights the implementation limitation of a binary biomarker scheme that leaves many patients unclassified.

For immunoparalysed patients, the therapeutic logic is different, but the supporting studies vary substantially in design and evidentiary strength. A biomarker-guided, double-blind randomized trial of GM-CSF enrolled 38 patients with persistently low monocyte HLA-DR and was designed primarily to test reversal of monocyte deactivation; it demonstrated immune restoration but was not powered to establish a mortality benefit (89). The frequently cited interferon-gamma report was an uncontrolled restoration study in nine septic patients with low monocyte HLA-DR, showing recovery of monocyte antigen presentation and cytokine production rather than randomized evidence of clinical efficacy (90). The IRIS-7 randomized phase 2 trial enrolled patients with septic shock and severe lymphopenia; intramuscular recombinant IL-7 increased absolute lymphocyte and CD4/CD8 T-cell counts and was generally tolerated, but its principal aims were safety and reversal of lymphopenia rather than mortality or organ-support outcomes (91). These interventions should therefore remain investigational and be evaluated within biomarker-guided trials.

Immune checkpoint pathways such as PD-1/PD-L1 and CTLA-4 are conceptually attractive because T-cell exhaustion may contribute to persistent or secondary infection, but checkpoint modulation remains investigational and should not be presented as a clinical recommendation (72, 73). Evidence for intravenous immunoglobulin and other antibody-based therapies also remains heterogeneous. A recent systematic review of 29 randomized trials found substantial variation in antibody targets, products, populations, and study quality, preventing a single class-wide conclusion (92). Although some meta-analyses and observational studies report mortality signals for standard or IgM-enriched immunoglobulin, the 2026 Surviving Sepsis Campaign suggests against routine IV immunoglobulin use because the certainty of evidence remains low (6).

The safest current position is that immunomodulation in sepsis and septic shock is moving from empiric broad application toward biomarker-guided precision therapy, but most interventions require prospective validation before routine use. Precision immunomodulation requires at least four conditions: a reproducible immune-state classifier, a therapy matched to the implicated mechanism, timing aligned with disease trajectory and source control, and dynamic monitoring of biological and clinical response. ImmunoSep advances the field by demonstrating prospective enrichment, while its neutral mortality result, safety signals, and large unclassified screening population show why a single binary framework is not yet sufficient (30, 77).

## 8 Bedside precision tools

Precision care must be operational at the bedside. The objective is not to accumulate measurements, but to combine rapidly available, repeatable, and physiologically interpretable information that changes a clinical decision. The tools in this section complement the mechanistic discussions of fluid therapy, vasopressor therapy, and biological endotyping in Sections 4–6.

Table 2 summarizes point-of-care ultrasound applications for hemodynamic phenotyping, whereas Table 3 presents complementary non-ultrasound clinical and biological tools. The tables are separated by modality and purpose to avoid duplicating the same assessments.

**Table 2:** Bedside point-of-care ultrasound applications for precision hemodynamic phenotyping in septic shock.

POCUS application	Precision question	Clinical implication
LVOT-VTI or stroke-volume estimation	Is forward flow low, preserved, or improving?	Distinguishes predominantly vasoplegic shock from low-output or myocardial dysfunction phenotypes.
Passive leg raising with VTI or stroke-volume assessment	Is the patient preload responsive?	Supports fluid administration only when a meaningful increase in forward flow is demonstrated.
Right ventricular size and systolic function	Is right ventricular dysfunction contributing to shock?	Discourages indiscriminate fluid loading and prompts reassessment of ventilation, pulmonary vascular load, and vasoactive strategy.
Lung ultrasound B-lines	Is pulmonary interstitial congestion present?	Suggests reduced fluid tolerance and raises the threshold for additional volume expansion.
Venous Doppler congestion assessment/VExUS	Is systemic venous congestion present?	Supports fluid restriction, consideration of deresuscitation after stabilization, or ultrafiltration in selected patients.

**Table 3:** Complementary non-ultrasound bedside and biological tools for precision septic shock care.

Tool	Practical availability	Precision decision supported
Serial lactate assessment	Widely available	Tracks metabolic and perfusion trajectory; persistent elevation should prompt reassessment rather than reflexive fluid escalation.
Capillary refill time	Universally available	Provides rapid bedside assessment of peripheral perfusion response.
Monocyte HLA-DR	Specialized or academic-center testing	Supports immune stratification, particularly for immunoparalysis-focused clinical-trial eligibility.
Clinical immune-surrogate pattern	Universally observable but nonspecific	Persistent lymphopenia, recurrent or secondary infection, or impaired pathogen clearance may raise suspicion for immunosuppression when formal immune testing is unavailable.

Serial lactate trajectories are prognostically informative (93, 94), but persistent hyperlactatemia is not a mechanism-specific signal. It may result from ongoing global or regional hypoperfusion, adrenergic-driven aerobic glycolysis, impaired hepatic clearance, altered pyruvate metabolism, mitochondrial dysfunction, or combinations of these processes (19). Even after the target MAP has been achieved, endothelial swelling, microvascular shunting, microthrombosis, altered vascular reactivity, impaired capillary recruitment, or venous congestion may leave regional organ perfusion inadequate despite acceptable macrocirculatory variables (18, 52, 53, 68). Lactate should therefore be interpreted with capillary refill time, urine output, hemodynamics, source-control status, and ultrasound findings. Failure to clear lactate should trigger a search for the dominant mechanism rather than automatic fluid administration.

Renin is discussed in Section 5 as a mechanistically relevant marker of renin-angiotensin system dysregulation, circulatory stress, and possible angiotensin II responsiveness. At the bedside, serial renin may complement—not replace—lactate, capillary refill time, urine output, echocardiography, congestion assessment, vasopressor requirements, and organ-function trajectories; validated treatment thresholds are not yet available (63–65).

Capillary refill time is inexpensive, immediately repeatable, and linked to peripheral perfusion. ANDROMEDA-SHOCK showed that capillary-refill-time-targeted resuscitation was associated with less organ dysfunction at 72 hours compared with lactate-targeted care, although the primary mortality endpoint did not reach statistical significance (68). Capillary refill time is best used as a complement to lactate and macrocirculatory assessment, not as a replacement for clinical judgment.

The principles of fluid responsiveness and fluid tolerance are developed in Section 4. Point-of-care ultrasound operationalizes these concepts by characterizing forward flow, ventricular function, preload responsiveness, pulmonary congestion, and systemic venous congestion. The principal applications and their decision implications are summarized in Table 2 (33, 34, 95, 96). VExUS may support congestion assessment, but it is not yet a validated sepsis-specific treatment algorithm (96).

The biological rationale and limitations of immune and endothelial phenotyping are discussed in Section 6. When available, monocyte HLA-DR can support trial-oriented immunoparalysis stratification (76, 77), while syndecan-1, endocan, and other endothelial biomarkers currently provide prognostic information rather than validated treatment targets (23–25, 78). In resource-limited settings, precision care should not depend on expensive assays. Repeated clinical reassessment, capillary refill time, lactate trends, urine output, basic laboratory values, and focused ultrasound can still support phenotype-aware decision-making.

## 9 Pragmatic precision framework for clinical practice

The core question in precision septic shock care is not simply “What should be done?” but “What should be done for this patient at this time?” Early management still requires rapid antibiotics, source-control planning, initial hemodynamic stabilization, oxygenation and ventilation support, and organ support as indicated (6, 20). Precision begins immediately afterward by repeatedly reclassifying the dominant mechanism of shock and matching therapy accordingly.

A practical framework has five steps. First, stabilize immediately: recognize septic shock, obtain cultures when feasible without delaying treatment, administer antimicrobials, begin source-control planning, start hemodynamic support, and identify immediate life threats. Second, define the early phenotype: evaluate fluid responsiveness, fluid tolerance, cardiac function, vasoplegia, peripheral perfusion, lactate trajectory, organ dysfunction, and congestion. Third, match therapy: give limited fluid only when responsiveness and tolerance are plausible; start or escalate norepinephrine when vasoplegia predominates; consider adjunct vasopressors when catecholamine burden is high; evaluate inotropy when low output and hypoperfusion are documented; consider corticosteroids in persistent vasopressor-dependent shock; and reserve other immunomodulators for selected or trial-based contexts. Fourth, reassess frequently: MAP, capillary refill time, lactate kinetics, urine output, ultrasound findings, vasopressor dose, cumulative fluid balance, and source-control status should guide adjustment. Fifth, de-escalate when possible: reduce vasopressors, stop non-beneficial fluids, move toward deresuscitation when congestion dominates, and monitor for secondary infection or immunosuppression. The transition from protocol-guided stabilization to precision-oriented refinement is summarized in Table 4, and the overall workflow is depicted in Figure 3.

**Table 4:** Conceptual transition from initial protocol-guided septic shock care to precision-oriented refinements.

Clinical domain	Initial protocol-guided framing	Precision-oriented framing
Fluid administration	Guideline suggestion of at least 30 mL/kg IV crystalloid within the first 3 hours for sepsis-induced hypoperfusion or septic shock (6).	Use the initial recommendation as a population-level default, with repeated reassessment; subsequent fluid depends on perfusion, fluid responsiveness, fluid tolerance, and congestion.
Vasopressor target	Initial MAP target of approximately 65 mmHg (6).	Use 65 mmHg as a starting floor, then individualize according to tissue perfusion and organ response; avoid routine high targets in older patients.
Vasopressor strategy	Norepinephrine first-line; add vasopressin when norepinephrine requirements escalate (6).	Start norepinephrine early when vasoplegia predominates and use mechanistically distinct adjuncts selectively rather than as a routine simultaneous multimodal regimen.
Corticosteroids	Use in selected vasopressor-dependent shock	Consider intravenous corticosteroids for adults with ongoing vasopressor-dependent septic shock after adequate resuscitation; hydrocortisone 200 mg/day is the regimen most commonly represented in randomized trials (6, 85–87).
Immunomodulation	Empiric treatment or complete avoidance	Biomarker-informed and endotype-directed, but still largely investigational outside corticosteroid use and trial settings.
Deresuscitation	Often delayed or passive consideration	Actively considered once shock stabilizes and clinically meaningful congestion or positive fluid balance emerges.

Protocol-guided care remains essential for early recognition and initial stabilization. Precision-oriented refinements are intended to complement, not replace, time-sensitive antimicrobial therapy, source-control planning, and early hemodynamic stabilization, especially after the first 1–2 hours of management.

**Figure 3:**
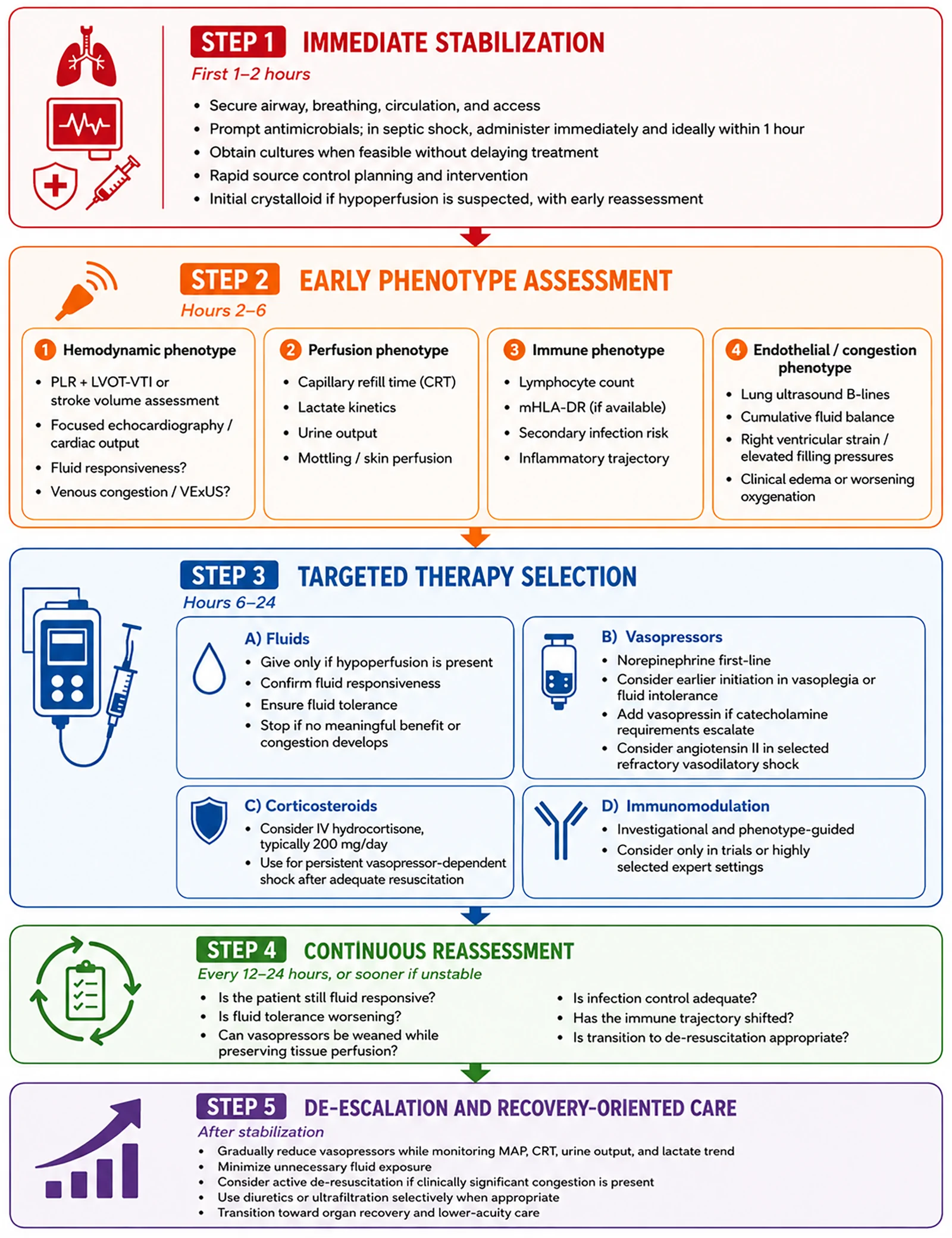
Precision resuscitation algorithm for septic shock. Proposed stepwise, phenotype- and endotype-guided framework for individualized septic shock management. The algorithm integrates five sequential but dynamically revisited phases: immediate stabilization, early multi-domain assessment, targeted therapy selection, continuous reassessment, and de-escalation toward recovery-oriented care. Early management prioritizes time-sensitive interventions, including antimicrobial therapy, source-control planning, and initial hemodynamic stabilization. Subsequent decisions are refined according to hemodynamic phenotype, tissue-perfusion status, immune trajectory, and evidence of endothelial dysfunction or fluid intolerance. Time windows are approximate and should be adapted to the patient's clinical evolution. CRT, capillary refill time; LVOT-VTI, left ventricular outflow tract velocity-time integral; MAP, mean arterial pressure; mHLA-DR, monocyte human leukocyte antigen-DR; PLR, passive leg raising; VExUS, venous excess ultrasound score.

This framework should not be misinterpreted. Precision medicine does not justify delaying antimicrobials or source control; withholding fluid from clearly hypoperfused, fluid-responsive patients; using investigational immunomodulators as routine care; or treating biomarkers as substitutes for clinical judgment (6, 15, 20, 76, 77, 88–91). It means stopping further escalation when physiology no longer supports benefit and adding targeted monitoring when the mechanism is unclear.

## 10 Future directions

Future septic shock trials should move beyond broad syndrome-level enrollment. Adaptive platform trials can evaluate multiple interventions, stop ineffective arms early, incorporate new therapeutic domains, and modify randomization as evidence accumulates (97, 98). This design is particularly relevant for therapies whose effects depend on timing, immune state, endothelial injury, infection source, or organ dysfunction.

Predictive enrichment should become central. Instead of enrolling all patients with septic shock into the same intervention pathway, trials should stratify by biologically plausible treatment-response markers: monocyte HLA-DR for immunoparalysis, hyperinflammatory cytokine or ferritin patterns for anti-inflammatory strategies, endothelial markers for vascular injury or fluid intolerance, renin or related markers for refractory vasodilatory shock, and integrated clinical phenotypes for fluid and vasopressor strategies (15, 60, 71, 76, 77).

Real-time immune profiling remains a major implementation barrier. For immune-guided therapy to influence clinical care, assays must be fast, standardized, affordable, interpretable, and linked to validated therapeutic pathways. The large unclassified population in ImmunoSep illustrates the limitations of relying on two thresholds alone (77), while recent consensus work suggests that continuous myeloid and lymphoid dysregulation scores may capture overlapping immune states more faithfully (30). Machine learning and multi-omics may improve endotype detection, but bedside translation will require prospective validation, calibration across populations, and evidence that test-guided treatment improves patient-centered outcomes (9, 10, 26, 27, 30, 71, 99).

Resource-sensitive implementation is equally important. Many ICUs cannot access advanced immune assays or microcirculatory imaging. Precision care in such settings should emphasize high-value bedside tools: repeated perfusion assessment, lactate kinetics, urine output, fluid balance, basic laboratory trends, focused ultrasound when available, and disciplined source-control reassessment. Precision medicine should narrow, not widen, the gap between guideline ideals and real-world practice.

## 11 Conclusion

Septic shock should be treated urgently but not simplistically. It is a dynamic syndrome in which patients with the same clinical diagnosis may differ in hemodynamic phenotype, fluid responsiveness, fluid tolerance, endothelial injury, immune state, microcirculatory function, and treatment responsiveness. Precision management does not replace guideline-based care; it refines it through repeated physiological interpretation.

Fluid therapy should be titrated according to both responsiveness and tolerance. Vasopressor therapy should move beyond a uniform MAP target toward assessment of tissue perfusion, mechanism of hypotension, catecholamine burden, and individualized weaning. Immune and endothelial phenotyping can help explain heterogeneity and enrich future trials, but most immunomodulatory strategies remain investigational outside established corticosteroid use in selected vasopressor-dependent shock. The practical future of septic shock care lies in combining time-critical treatment with bedside phenotyping, dynamic reassessment, and biologically informed therapeutic matching.
